# Preliminary In Vitro and In Vivo Insights of In Silico Candidate Repurposed Drugs for Alzheimer’s Disease

**DOI:** 10.3390/life13051095

**Published:** 2023-04-27

**Authors:** Kyriaki Savva, Margarita Zachariou, Demos Kynigopoulos, Eleni Fella, Maria-Ioanna Vitali, Xeni Kosofidou, Michail Spyrou, Irene Sargiannidou, Elena Panayiotou, Nikolas Dietis, George M. Spyrou

**Affiliations:** 1 Bioinformatics Department, The Cyprus Institute of Neurology and Genetics, 2371 Nicosia, Cyprus; kyriakis@cing.ac.cy (K.S.); margaritaz@cing.ac.cy (M.Z.); 2 Department of Neuropathology, The Cyprus Institute of Neurology and Genetics, 2371 Nicosia, Cyprus; kynigopoulosd@cing.ac.cy (D.K.); elenife@cing.ac.cy (E.F.); 3 Experimental Pharmacology Laboratory, Medical School, University of Cyprus, 2109 Nicosia, Cyprus; vitali.maria-ioanna@ucy.ac.cy (M.-I.V.); kosofidou.xeni@ucy.ac.cy (X.K.); spyrou.michail@ucy.ac.cy (M.S.); 4 Neuroscience Department, The Cyprus Institute of Neurology and Genetics, 2371 Nicosia, Cyprus

**Keywords:** Alzheimer’s disease, in vitro testing, in vivo testing, in silico drug repurposing

## Abstract

Alzheimer’s disease (AD) is a progressive neurodegenerative disease and is the most common type of dementia. Although a considerably large amount of money has been invested in drug development for AD, no disease modifying treatment has been detected so far. In our previous work, we developed a computational method to highlight stage-specific candidate repurposed drugs against AD. In this study, we tested the effect of the top 13 candidate repurposed drugs that we proposed in our previous work in a severity stage-specific manner using an in vitro BACE1 assay and the effect of a top-ranked drug from the list of our previous work, tetrabenazine (TBZ), in the 5XFAD as an AD mouse model. From our in vitro screening, we detected 2 compounds (clomiphene citrate and Pik-90) that showed statistically significant inhibition against the activity of the BACE1 enzyme. The administration of TBZ at the selected dose and therapeutic regimen in 5XFAD in male and female mice showed no significant effect in behavioral tests using the Y-maze and the ELISA immunoassay of Aβ40. To our knowledge, this is the first time the drug tetrabenazine has been tested in the 5XFAD mouse model of AD in a sex-stratified manner. Our results highlight 2 drugs (clomiphene citrate and Pik-90) from our previous computational work for further investigation.

## 1. Introduction

Alzheimer’s disease (AD) is a progressive neurodegenerative disease and is the most common type of dementia. Over the past decade, great effort has been put into understanding the pathogenesis and mechanisms involved in neurodegenerative diseases, as well as discovering novel treatments for these diseases. Although a considerably large amount of money has been invested in drug development for AD, only five symptomatic treatments have been approved so far (i.e., donepezil, galantamine, memantine, rivastigmine, and combination of memantine and donepezil) [1], along with the recently FDA approved drug lecanemab, which was granted an accelerated approval and needs further investigation [2].

According to the amyloid hypothesis, β-secretase (BACE1; β-site amyloid precursor protein cleaving enzyme 1), an enzyme triggering the formation of the Aβ peptide, is a key drug target to be exploited for the development of novel anti-AD drugs [3]. This is supported by studies showing that germline deletion of BACE1 halts Aβ production and improves cognitive and behavioral abilities, observed in transgenic mice [4,5]. Moreover, a mutation at the BACE1 cleavage site of amyloid precursor protein (APP) causes a decrease in Aβ production by half [6,7]. All of the aforementioned information—as well as several other reasons—makes BACE1 a great candidate target for treating AD patients. However, BACE1 inhibition has also faced criticism recently, due to the high rate of failure of BACE1 inhibitors in the clinical trials. A recent study by Chatila et al. (2018) suggested that partial, rather than full inhibition of BACE1, may be a better therapeutic strategy for AD since complete loss of BACE1 activity has devastating effects on adult hippocampal neurogenesis [8].

Although a large number of AD-related drugs have entered the preclinical phase, the failure rate has been very high due to the complexity of the disease [9]. Various AD-related drugs have failed in clinical trials yet, currently, there are still 566 interventions in clinical trials under the recruiting phase (clinicaltrials.gov). In order to identify potential novel treatments for such a complex disease, an alternative approach can be applied, known as drug repurposing or repositioning [10], which is the identification of novel indications for currently-used drugs [11]. A major advantage of the drug repurposing strategy is that it involves compounds, with a known pharmacokinetic profile, which have already been approved through preclinical safety testing and therefore, repositioned drugs render the clinical phase process much faster than newly developed drugs, in a more cost-effective way, with a reduced risk of failure. Hence, by using existing pharmacokinetic, toxicology and safety data, this drug repurposing substantially reduces the risks related to the traditional de novo discovery of drugs [12].

In our recent work [13], we developed a novel network-based computational method to propose stage-specific candidate repurposed drugs against AD. For each AD stage, we ranked the candidate repurposed drugs based on their structural similarity with failed, approved or currently in clinical trials drugs for AD, based on other related functional and structural information as well as based on permeability prediction through the Blood Brain Barrier (BBB). In that study, we highlighted and proposed 26 candidate repurposed drugs across the 3 stages of AD stages for experimental validation.

The aim of this study was to test experimentally in vivo and in vitro the top candidate repurposed drugs as proposed in our previous work. We examined the effects of the top five drugs per AD stage (13 drugs in total) derived from our previous computational drug repurposing work [13], against the enzymatic activity of the BACE1 enzyme, using a commercial inhibitor screening assay.

Moreover, we simultaneously tested the top drug detected in Braak stage V–VI from our computational study [13], Tetrabenazine (TBZ). Interestingly, TBZ is an FDA approved drug for Huntington’s disease (HD), another disease under the umbrella of neurodegeneration [14,15]. TBZ, a potent inhibitor of the transporter of vesicular monoamines (such as dopamine, serotonin, norepinephrine, and histamine) and transporter VMAT2 [16] was chosen to be tested in this animal model because it is a chemical compound that has not been previously tested in AD models. The effects of TBZ were examined in the 5XFAD animal model, by testing the behavioral aspects of female and male mice using the Y-maze and by measuring the Aβ40 levels using ELISA. Two transgenes are expressed in this animal model: the human *APP* and *PSEN1* transgenes. These transgenes are accompanied by a total of 5 familial AD related mutations, known as the Swedish (K670N/M671L), Florida (I716V), and London (V717I) mutations in *APP*, and the M146L and L286V mutations in *PSEN1*. The 5XFAD mouse model recapitulates a number of different AD-related phenotypes and shows a relatively early and aggressive cerebral amyloidosis phenotype. At about two months of age, amyloid plaques, along with gliosis can be detected. At about six months of age, neuronal loss occurs in multiple regions of the brain. Moreover, mice exhibit a range of cognitive and motor deficits [17].

## 2. Materials and Methods

**Figure 1:**
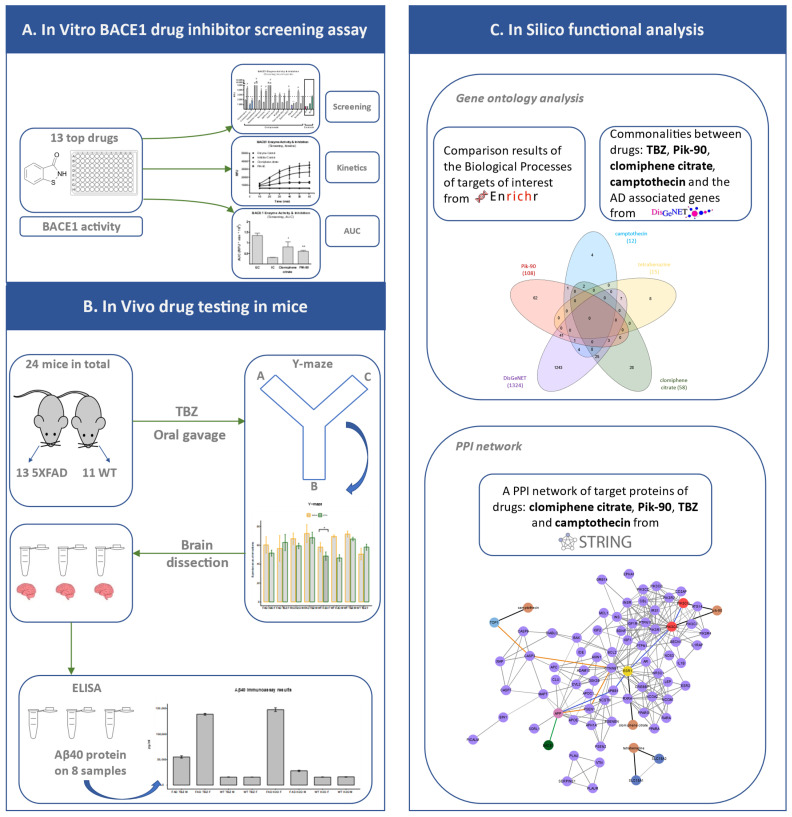
Overview of the workflow. (**A**) In vitro BACE1 drug inhibitor screening assay: the top 13 candidate repurposed drugs detected from our previous computational work were tested against the BACE1 enzyme activity. The symbol # depicts the compounds that showed increased enzymatic activity. (** *p* < 0.005, * *p* < 0.05). (**B**) In vivo drug testing in mice: the effect of tetrabenazine (TBZ) was tested in a total of 24 male and female mice, both wild-type and 5XFAD. 13 5XFAD mice and 11 WT mice. TBZ was delivered to the mice via oral gavage as previously described [18]. Next, Y-maze test was performed before treatment and at the end of treatment, 3 months later. (* *p* < 0.05). After completion of behavioral tests, mice were anesthetized and brains were dissected. Lastly, ELISA was performed on 8 of the 24 samples. (**C**) In silico functional analysis: comparison results of the Biological Processes of targets of interest. Commonalities in “biological processes” using GO that are targeted by TBZ, Pik-90, clomiphene citrate, camptothecin (that showed enhanced effects on the BACE1 assay) and the AD-associated genes from DisGeNET are shown. A protein-to-protein network was generated using the target proteins of clomiphene citrate, Pik-90, TBZ and camptothecin.

The workflow of the proposed pipeline in this paper can be described in the following main steps (as illustrated in detail in Figure 1):

In the first part (panel A), we ran an in vitro BACE1 drug inhibitor screening assay: the top 13 candidate repurposed drugs detected from our previous computational work were tested against the BACE1 enzyme activity. This assay was performed in order to detect which drugs show an effect on BACE1 enzyme. Kinetics and AUC of the two chemical compounds that showed statistically significant inhibition of BACE1 are shown. In the second part (panel B), we conducted in vivo drug testing in mice: the effect of tetrabenazine (TBZ), the candidate repurposed drug detected with the highest score for the stage of severe AD (Braak stage V–VI) of our previous work [13], was tested in a total of 24 male and female mice, wild-type and 5XFAD, 13 5XFAD mice (8 of them control and 5 took TBZ), and 11 WT mice (5 control and 6 TBZ). In total, 11 male and 13 female mice were used. TBZ was delivered to the mice via oral gavage as previously described [18]. This was administered to the mice three times a week for a total of three months, starting at two months of age. Next, working memory and exploratory ability were measured using the Y-maze test, which was performed before treatment and at the end of treatment, 3 months later. After completion of behavioral tests, mice were anesthetized and brains were dissected. Lastly, ELISA was performed on 8 of the 24 samples. In the last part (panel C), we ran the bioinformatics functional analysis: gene ontology (GO) analysis was performed using Enrichr [19]. We then searched for commonalities in GO “biological processes” that are targeted by TBZ, Pik-90, clomiphene citrate, camptothecin, and the AD-associated genes from DisGeNET are shown. Lastly, a protein-to-protein network was generated using the target proteins of clomiphene citrate, Pik-90, TBZ and camptothecin through STRING v11.5 database [20].

### 2.1. In Vitro BACE1 Inhibitor Screening Assay

BACE1 enzyme activity was measured for the selected candidate repurposed drugs listed in Table 1, using the BACE1 inhibitor screening kit from Abcam (ab283408) (Cambridge, UK), according to the manufacturer’s instructions.

Briefly, the chemical compounds were diluted in 10% DMSO to get a final concentration of 100 μM in the well. To measure the activity and inhibition of BACE1 enzyme, 50 μL of the sample were mixed with 2 μL of BACE1 enzyme and 50 μL of substrate solution in a 96-well white plate with a flat transparent bottom. The assay was carried out using 3 replicates per compound. The controls used were the enzyme control (EC) that contained only enzyme, the inhibitor control (IC) that contained a comparative assay inhibitor, and the solvent control (SC) that contained only the solvent included in the compound solutions. The enzyme’s activity was observed through fluorescent intensity measured for 1 h at intervals of 10 min using excitation 345 nm and emission 500 nm at 37 °C according to the manufacturer’s protocol. Two time points were chosen in the linear range of the produced plot to obtain the corresponding values for the relative fluorescence units (RFU). Calculations of % relative inhibition scores and % relative activity were made according to the manufacturer’s instructions (Calculation A and B).

$$Relative\;Activity\;\left(\%\right)=\frac{Slope\;of\;S}{Slope\;of\;EC}\times 100\;(\mathrm{Calculation}\;A)$$

$$Relative\;inhibition\;\left(\%\right)=\frac{Slope\;of\;EC-Slope\;of\;S}{Slope\;of\;EC}\times 100\;(\mathrm{Calculation}\;B),$$

where *Slope of EC* is the slope of enzyme control and *Slope S* is the slope of sample screen.

### 2.2. In Vivo Testing in Mice

#### 2.2.1. Drug Delivery in 5xFAD Mice

For this study, the effect of TBZ, the repurposed candidate drug detected with the highest score for the severe AD stage (Braak stage V–VI) from our previous work [13] was tested in male and female mice, both wild-type and 5XFAD. Twenty-four mice in total were used for our experiment: 13 5XFAD mice (8 of them control and 5 took TBZ) and 11 WT mice (5 control and 6 TBZ). In total, 11 male and 13 female mice were used. TBZ (100 mg) was obtained from Cayman Chemical (Ann Arbor, MI, USA), mixed with 2% corn-flour using a ceramic grinder and re-suspended in phosphate-buffered saline (PBS). TBZ was delivered to the mice via oral gavage as previously described [18]. It was administered to the mice three times a week for a total of three months, starting at two months of age. Mice were fed 0.125 mg of TBZ suspended in 50 μL of PBS with 2% corn flour. All control mice had only H_2_O in their diet.

#### 2.2.2. Y-Maze Test in 5xFAD Mice

Working memory and exploratory ability were measured using the Y-maze spontaneous alternation protocol. Y-maze is a Y-shaped compartment with equal length arms. These arms are labelled with the letters A, B, and C. Each mouse was initially placed in the central area while allowed to freely explore the three maze arms. The number of entries into the arms and alterations were recorded for 6 min. Working memory was calculated as the number of correct alterations/numbers of total new arm entries, as described by [21]. Y-maze test was performed before treatment and at the end of treatment, 3 months later.

#### 2.2.3. Brain Dissection of 5xFAD Mice

After completion of behavioral tests, mice were anesthetized with Avertin and transcardially perfused with 5 mL of 0.9% saline followed by 5 mL of fixative (4% paraformaldehyde in 0.1 M PBS, pH 7.4). All brains were removed from the skull, weighed, and transferred to post-fixative overnight at 4 °C in 4% paraformaldehyde and equilibrated in 20–30% (*w*/*v*) sucrose in PBS. At sacrifice, the brains of the mice were divided by mid-sagittal dissection, and one hemisphere was used for biochemical analysis (ELISA assay) and the other was fixed for later use.

#### 2.2.4. ELISA

The enzyme-linked immunosorbent assay (ELISA) was performed on 8 of the 24 samples—the ones that showed promising results in the Y-maze test. Samples were used in three technical replicates. The hemispheres of the sacrificed mice were weighted (~100 mg) and lysed using 8x the brain mass of cold 5M guanidine-HCl/50 mM Tris. The samples were then homogenized by sonication. The lysate was centrifuged at 16,000× *g* for 20 min at 4 °C. The supernatant was collected to evaluate the amount of Aβ40 (pg/mg protein) following the manufacturer’s protocol (KMB3481, Thermo Fisher Scientific, Waltham, MA, USA).

### 2.3. Statistical Analysis

Data are presented as standard deviation of the means with Bonferroni multiple comparisons for the in vitro tests. The normality of all datasets was tested using the Shapiro-Wilk test. Y-maze test differences between the same mice (before and after) were determined by paired *t*-test. Differences in the behavioral test between different groups were determined by a non-paired *t*-test. ELISA statistical analysis was performed using unpaired Wilcoxon test. R statistical environment 4.2.3 (http://www.R-project.org/, Indianapolis, IN, USA) and Prism software (Irvine, CA, USA) were used for statistical analyses and generation of graphs, and *p* ≤ 0.05 was considered statistically significant. Different statistical tests used in this study are summarized in Table 2.

### 2.4. Bioinformatics Functional Analysis

#### 2.4.1. Gene Ontology Analysis

Gene ontology (GO) analysis was performed through Enrichr [19] using the EnrichR package in R. Drug targets of each of the drugs of interest were used as input; *ESR1* for clomiphene citrate, *PIK3CG* and *PIK3CA* for Pik-90, *SLC18A1* and *SLC18A2* for TBZ, *TOP1* for camptothecin and the top scored genes associated to AD from DisGeNET. DisGeNET is a platform that contains one of the largest publicly available collections of genes associated with human diseases [22]. A threshold of gene-disease association (gda) score ≥0.5 was used in order to keep the most important genes associated to AD. Membership was obtained using “GO Biological Process 2021” as the database partition.

#### 2.4.2. PPI Network Construction

The associations of the investigated proteins were analyzed using the STRING v11.5 database [20] (http://www.string-db.org, accessed on 1 December 2022), which integrates information about the interaction of proteins (obtained as a result of experimental studies and curated databases) into a single network. The required confidence was set to >0.7 as the threshold for protein-protein interaction to construct the network. In addition, Cytoscape (Institute for Systems Biology, Seattle, WI, USA) [23] was used to visualize the network.

## 3. Results

### 3.1. Effect of Tested Compounds on BACE1 Enzyme Activity

**Figure 2:**
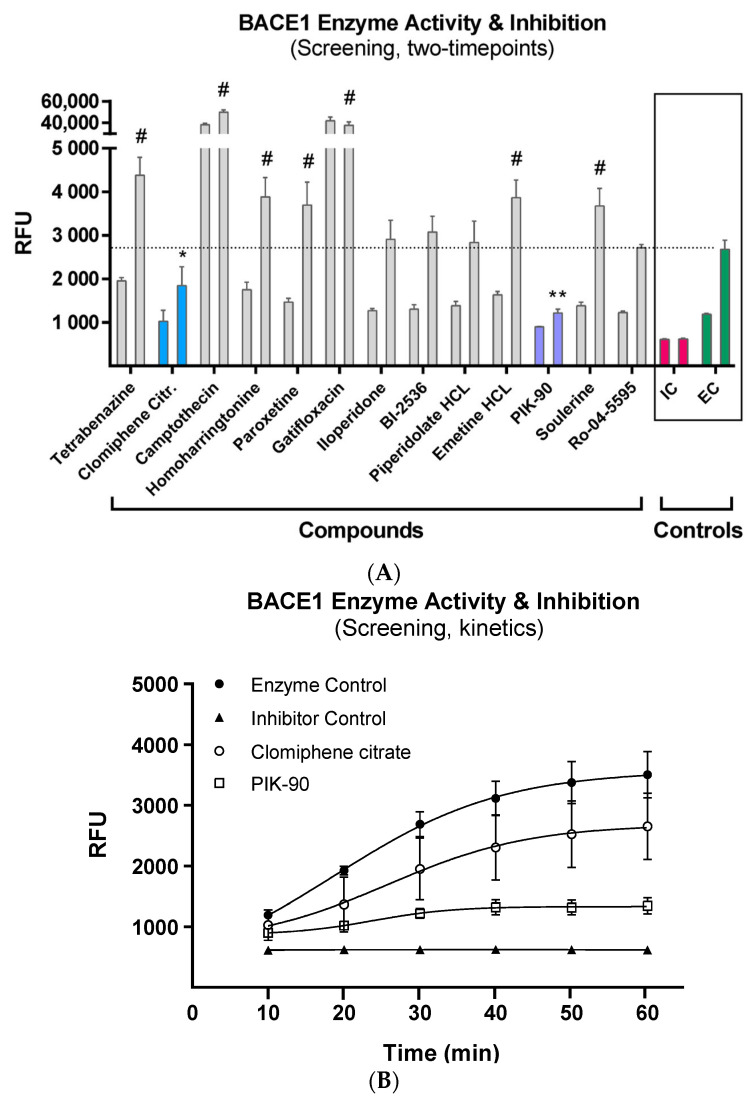
BACE1 inhibitor assay kinetics and screening. (**A**) Panel A shows the maximum activity of the chemical compounds in the BACE1 inhibitor assay, compared to the inhibitor control and enzyme control. Two time points are shown here for each drug, at 10 min and 30 min. The colored bars show the compounds that were statistically significant, using a One-Way ANOVA with Bonferroni multiple comparison. The symbol # depicts the compounds that showed increased enzymatic activity. (**B**) Panel B shows the kinetics of BACE1 enzyme inhibition of the top two compounds tested (clomiphene citrate and Pik-90) with the internal inhibitor control and enzyme control. (** *p* < 0.005, * *p* < 0.05).

We first examined the effect of 13 compounds at 100 μM final concentration of 100 M against the activity of the BACE1 enzyme using a standardized and pre-validated time-resolved fluorometric inhibitor assay. Data from two time points in the linear range (10 min and 30 min) were used to confirm enzyme inhibition, indicated by reduced relative fluorescence (Figure 2A). Two compounds (clomiphene citrate and Pik-90) showed significant enzyme inhibition at 30 min after treatment, compared to EC, whereas 7 compounds showed increased enzymatic activity (TBZ, homoharringtonine, camptothecin, paroxetine, gatifloxacin, emetine hydrochloride hydrate, and scoulerine). Compared to the internal IC, Pik-90 was the only one that showed no significant difference in inhibition 30 min after treatment.

We then examined clomiphene citrate and Pik-90 in a kinetic mode, measuring enzymatic activity from 10 to 60 min after treatment (Figure 2B). The linear range of the enzymatic activity was confirmed to be between 10 and 30 min, while the maximum activity was saturated between 50 and 60 min after the initiation of the reaction. The internal control produced complete inhibition for the entire test time. The inhibition profile of clomiphene citrate followed a slope similar to that of the enzyme control, while Pik-90 reached maximum inhibition at 30 min, which was sustained until the end of the assay.

**Figure 3:**
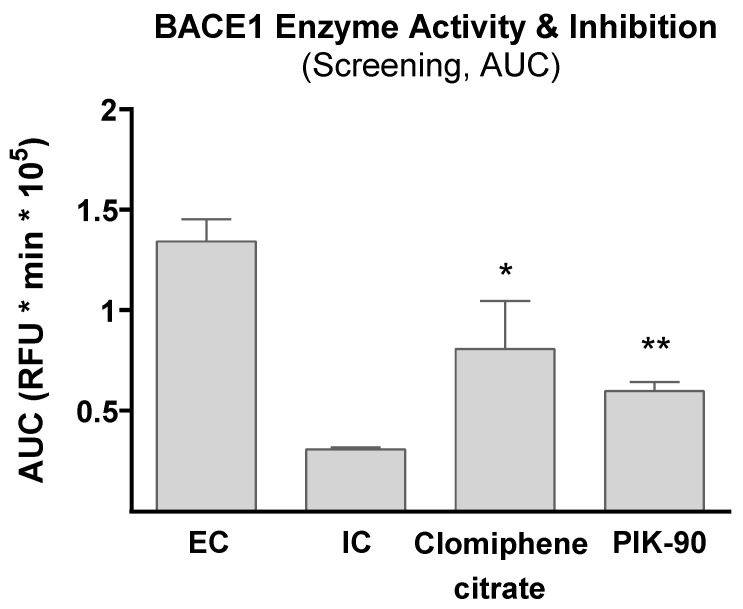
BACE1 assay AUC means. Overall inhibition of the 2 chemical compounds that showed statistically significant inhibition of BACE1 for the whole-time span (60 min). Statistical significance of the difference in fluorescence readout was calculated using a One-Way ANOVA with Bonferroni multiple comparison against EC (** *p* < 0.005, * *p* < 0.05).

In order to calculate the overall BACE1 inhibition of 100 μM clomiphene citrate and Pik-90, we calculated the area under the curve (AUC) of each curve in the kinetic assay. Both compounds showed significant overall inhibition in the course of 60 min after treatment, compared to enzyme control (Figure 3).

From the 13 list of the computationally highlighted drugs, we had the opportunity to select a single drug for a small-scale in vivo experiment. This was the drug TBZ, as it was top-scored for severe AD stage in our computational analysis even though it did not show a statistically significant inhibition in BACE1 enzyme activity.

### 3.2. Y-Maze Test before and after Tetrabenazine Treatment in 5XFAD Mice

**Figure 4:**
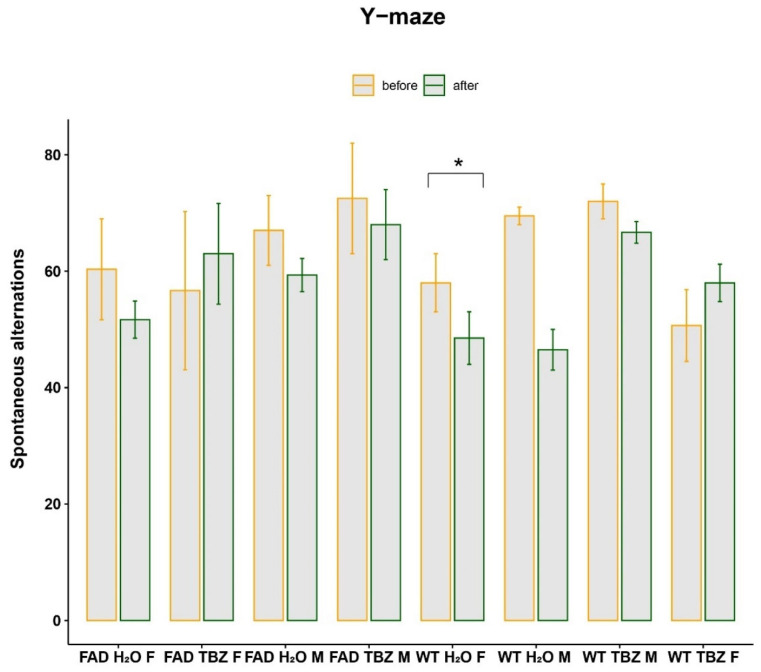
Y-maze test before and after treatment. The average percentage of correct alterations was measured in 5XFAD and WT mice, both treated with TBZ and water. The error bars show the standard deviation. Data were analyzed using a paired (between the same group) and non-paired *t*-test (between different groups). M = males; F = females (* *p* < 0.05).

Working memory was examined using the Y-maze spontaneous alternation test in treated and untreated 5XFAD and WT mice (balanced male/female cohort). The average percentage of correct alterations was significantly lower (*p*-value = 0.03) in WT female mice treated with water before and after three months. However, none of the other comparisons exhibited any statistical significance (Figure 4). These results suggest that TBZ treatment did not have a significant effect on the behavioral phenotype present in 5XFAD mice.

### 3.3. ELISA Test of Aβ Peptide in 5XFAD Mice

**Figure 5:**
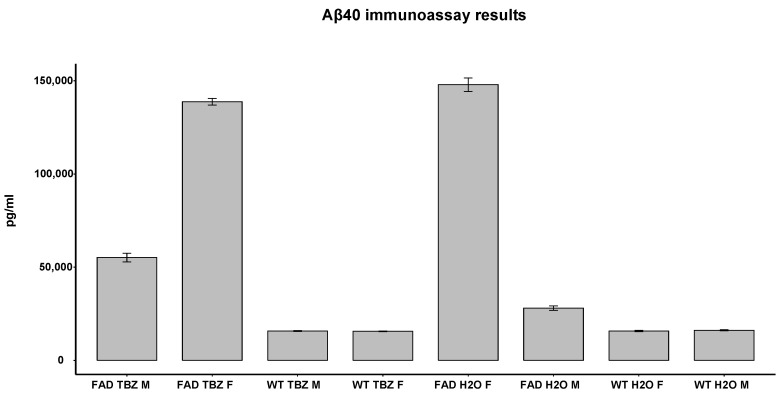
ELISA. Amyloid β levels were measured in 5XFAD and WT mice, both treated with TBZ and water. Mouse Aβ40 levels were detected by a mouse ELISA kit. Samples were used in three technical replicates. The error bars show the standard deviation. Data were analyzed using a unpaired Wilcoxon test. M = males; F = females.

ELISA was performed on 8 of the 24 samples as described in the Section 2. Using the Shapiro–Wilcox test, we detected that our data were not following the normal distribution. Therefore, the unpaired Wilcoxon test was carried out for statistical analysis. By measuring the Aβ40 levels in mice brains, none of the comparisons exhibited any statistical significance (Figure 5). However, for this analysis, only eight samples were used, and therefore, these results cannot lead to any conclusions. Although these results are not significant, we can observe some trends in our results, i.e., that the Aβ40 levels in 5XFAD male mice treated with TBZ are shown to be lower when compared to 5XFAD male mice treated with water. Moreover, 5XFAD females treated with water showed higher Aβ levels compared to 5XFAD males treated with water in the first place, as shown in the literature, suggesting that sex differences exist in the production of Aβ levels [17,24,25]. In addition, a trend that Aβ40 levels are decreased in treated females is observed. TBZ is shown to have inverse effects in males and females, perturbing in the correct direction the female mice.

### 3.4. Bioinformatics Functional Analysis

#### 3.4.1. Gene Ontology Analysis

**Figure 6:**
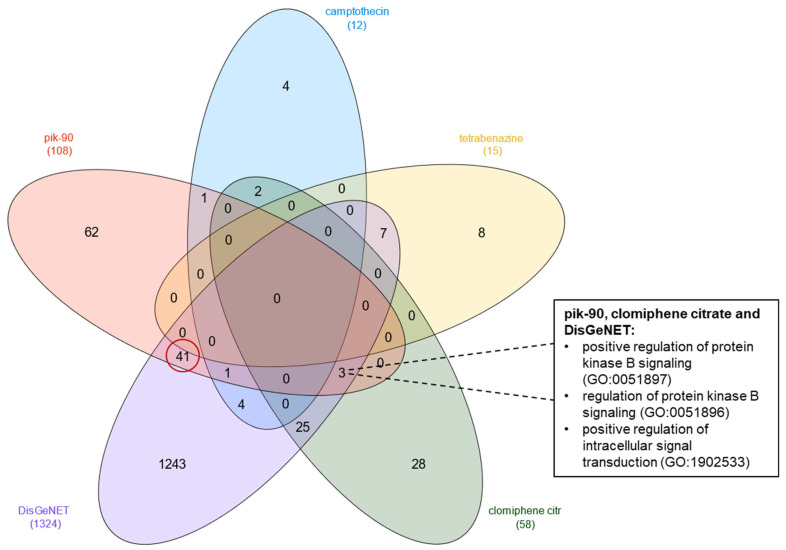
Comparison results of the biological processes of targets of interest. Commonalities in “biological processes” using GO that are targeted by TBZ, Pik-90, clomiphene citrate, camptothecin, and the AD-associated genes from DisGeNET are shown. The three common “biological processes” among clomiphene citrate, Pik-90 and AD-associated genes are shown. The commonalities between Pik-90 and DisGeNET genes are shown in a red circle highlighting the highest number of commonalities in our comparison analysis. The full list of “biological processes” can be found in Table S1.

Following the detection of the two drugs that showed effect using the BACE1 inhibitor assay, we carried out functional analysis of their targets. This post-analysis was performed to detect commonalities between the drugs investigated. For this analysis, we used clomiphene citrate and Pik-90, the 2 hits of the in vitro assay, the single drug tested in mice, TBZ, the drug camptothecin that showed enhanced effects on the BACE1 assay, and the AD-associated genes from DisGeNET. Firstly, we detected the “biological processes” related to the drug targets and genes from DisGeNET using EnrichR. From the “biological processes” detected, the noticeable results are the detection of three commonalities among Pik-90, clomiphene citrate, and the AD-associated genes; additionally, the highest number of commonalities (41) were detected between Pik-90 and the AD-associated genes from DisGeNET. Remarkably, Pik-90 was the drug that showed the strongest inhibition using the in vitro assay (Figure 6). The three common “biological processes” among Pik-90, clomiphene citrate and the AD-associated genes are the “positive regulation of protein kinase B signaling (GO:0051897)”, “regulation of protein kinase B signaling (GO:0051896)” and the “positive regulation of intracellular signal transduction (GO:1902533)”. A study by He et al. (2016) showed that activation of the PI3K/Akt pathway downregulates BACE1 action and therefore improves cognitive impairment in AD animal models [26]. Furthermore, in a study by Rosenberger et al. (2015), protein kinase activity profiling was performed in order to investigate changes in protein kinase activity with respect to AD progression, and a general decrease in protein kinase activity was found to be correlated with disease progression [27]. Interestingly, this decrease was detected in early stages of AD (Braak stages I–II), meaning that such biological events should be targeted early in the pathogenesis of AD. Moreover, the 2 hits we detected in the in vitro assay, clomiphene citrate and Pik-90, are drugs that were detected in the early stage of AD (Braak I–II) in our previous computational work [13].

#### 3.4.2. PPI Network Generation

To further analyze the functional aspects of the detected drugs and identify possible mechanisms behind them, we generated a protein–protein interaction (PPI) network using the three aforementioned drug targets, as well as the genes most associated with AD according to DisGeNET (including BACE1). This was carried out in order to detect any interactions between the targets of the assays and the targets of the drugs. The network was constructed using the STRING database [20], using *Homo sapiens* as the organism of interest; furthermore, it was expanded by a maximum of 20 additional interactors in the first shell and a maximum of 25 additional interactors in the second shell. The confidence cut-off for the interactions between the proteins was set at 0.7. The network was visualized in Cytoscape [23].

**Figure 7:**
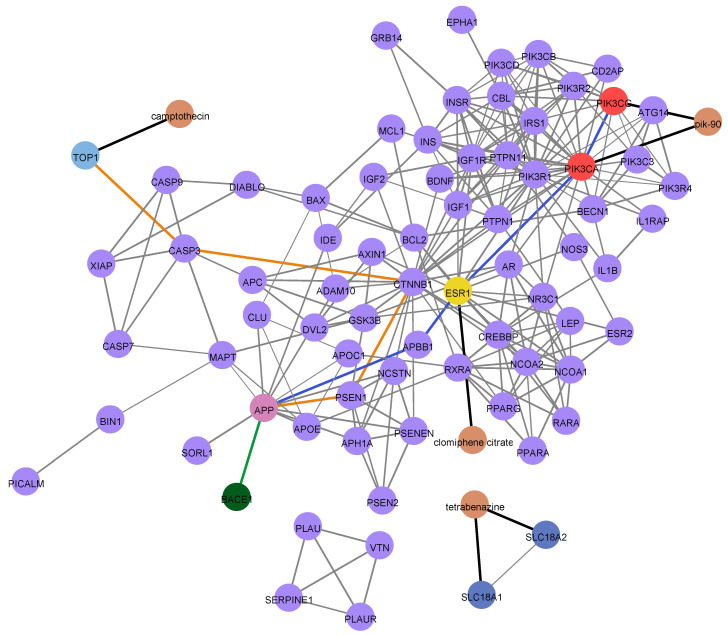
PPI network of target proteins of compounds under investigation. A protein-to-protein network was generated using the target proteins of clomiphene citrate, Pik-90, TBZ, and camptothecin. These drugs are represented in orange-colored nodes. Purple nodes represent the AD-associated proteins from DisGeNET, along with the first and second neighbors of the protein targets of interest. Blue-colored edges show the path that the targets of interest interact with the targets under question; BACE1 and APP are depicted as dark-green- and pink-colored nodes, respectively. The black edges show the drug–protein interaction. Orange-colored edges show the path that the target of camptothecin interacts with APP and BACE1, whereas the green-colored edges depict the common path of Pik-90, clomiphene citrate, and camptothecin. Blue nodes show the target proteins of TBZ, yellow node show the target protein of clomiphene citrate, red nodes show the target proteins of Pik-90, and light blue nodes the target of camptothecin.

The resulting PPI network using the drug target proteins as well as the protein targets of the in vitro assay are depicted in Figure 7. The drugs that target these proteins are colored orange. The purple nodes represent the first and second neighbors of the target proteins. Interestingly, both the target proteins of clomiphene citrate and Pik-90 interact with BACE1 and APP through some intermediate interactors. ESR1, the target protein of clomiphene citrate, interacts with both APP and BACE1 through the intermediate player APBB1. Additionally, PIK3CA and PIK3CG, the targets of Pik-90, interact with APP and BACE1 through ESR1 and APBB1. Notably, the target proteins of TBZ, SLC18A1, and SLC18A2, are isolated and do not interact with any of the other proteins in the network. These results could suggest a pathway by which clomiphene citrate and Pik-90 cause inhibition of both the BACE1 and Aβ peptide in the 2 respective in vitro assays. This bioinformatics functional analysis enhances our knowledge regarding the drugs of interest and hence, further supports the experimental findings.

## 4. Discussion

In this study, our aim was to test the effect of the 13 top candidate repurposed drugs as proposed in our previous computational drug repurposing work [13] using a commercial BACE1 inhibitor screening assay. At the same time, the effect of TBZ, the repurposed candidate drug detected with the highest score for the severe AD stage (Braak stage V–VI) from our previous work [13] was tested in male and female mice, both wild type and 5XFAD. We identified 2 compounds that showed statistically significant inhibition of BACE1 enzyme: clomiphene citrate and Pik-90. In the animal model, behavioral tests were carried out using Y-maze before and after treatment, as well as an ELISA A40 immunoassay of Aβ40 at the end of treatment, both of which did not show significant results on the effectiveness of TBZ in AD. Moreover, TBZ did not show any significant inhibitory effects against BACE1, yet on the contrary, it showed increased enzymatic activity. To our knowledge, this is the first time the drug TBZ has been tested in the 5XFAD mouse model of AD in a sex-stratified manner.

Regarding the effect of tested compounds on BACE1 enzyme activity, 2 chemical compounds were found to have significant inhibition of the BACE1 enzyme: clomiphene citrate and Pik-90. Interestingly, the former is an FDA approved drug while the latter is an experimental drug. Clomiphene citrate is a drug used in anovulatory women to stimulate ovulation and treat infertility, as well as in women that suffer from polycystic ovary syndrome [28]. Pik-90, which showed the highest inhibition score, is a PI3K inhibitor [29]. To our knowledge, neither clomiphene citrate nor Pik-90 have been previously tested against the activity of the BACE1 enzyme for AD.

Clomiphene citrate belongs to the group of selective estrogen receptor modulators (SERMs) as other drugs, such as tamoxifen, raloxifene, bazedoxifene, ospemifene, etc. SERM includes a wide category of compounds which act both as partial agonists by selectively influencing a certain type of estrogen receptors and also as antagonists on other types of signaling systems (membranous/intracellular) normally associated with natural estrogens [28]. Even though there are no available studies on clomiphene and AD, raloxifene, which belongs to SERM, too, has previously been tested in some clinical trials for AD. For instance, a 2005 study [30] showed that when raloxifene is administered in a dose of 120 mg/day, it has a preventive effect resulting in the reduction of AD risk and MCI by 33% after 36 months of treatment. On the other hand, in 2015, Henderson et al. conducted a double blind randomized clinical trial on 42 women with mild to moderate AD who either received 120 mg raloxifene orally or a placebo, and showed that raloxifene did not have a significant effect on ADAS-Cog score (Alzheimer’s disease assessment scale) [31].

Interestingly, Pik-90 is a known PI3K inhibitor. The PI3K/Akt signaling pathway is involved in many functions in the brain, such as regulating survival, cell proliferation, growth, differentiation, motility, intracellular trafficking, and more sophisticated processes such as the extension of neurites (dendrites and axons) [32]. Its role in the brain is essential since it is crucial in maintaining synaptic plasticity and memory processes [33]. Moreover, this pathway has a well-known connection to AD, since it has a key role in the regulation of Tau hyper-phosphorylation. Activation of PI3K/Akt pathway is neuroprotective, by promoting GSK3β phosphorylation. This phosphorylation facilitates its degradation, leading to reduction of hyper-phosphorylation of Tau [34,35]. Therefore, theoretically, PI3K inhibitors are damaging to AD as they inhibit the protective effect of PI3K and promote GSK3β activity. However, a study by Chiang et al. has shown that inhibition of the PI3K pathway led to the rescue of memory loss caused by the Aβ peptide in Drosophila [36]. Hence, based on the above, a PI3K inhibitor like Pik-90 could have positive effects against AD, especially since this drug has also shown inhibitory effects of the BACE1 enzyme.

Notably, seven chemical compounds (TBZ, homoharringtonine, camptothecin, paroxetine, gatifloxacin, emetine hydrochloride hydrate, and scoulerine) were found to exhibit a statistically significant increase of the BACE1 enzyme activity, with camptothecin and gatifloxacin causing the highest enzymatic activity, enhancing the cleavage of APP protein. This could lead to increased production of Aβ peptide, and therefore may have a higher risk of developing Alzheimer’s disease. Camptothecin, a natural product of the stem wood of the Chinese tree, *Camptotheca acuminate*, selectively inhibits the nuclear enzyme DNA topoisomerase, type I. Camptothecin and several semisynthetic analogs showed antitumor activity [37]. Gatifloxacin is an antibiotic agent, which inhibits the bacterial enzymes DNA gyrase and topoisomerase IV. Studies have shown that gatifloxacin has caused delirium and psychosis in AD patients [38,39].

The ELISA experiment did not show significant results on the treatment with TBZ in male and female mice. Although our sample size was limited and no conclusions can be drawn, some trends can be observed. It can be observed that Aβ40 levels in 5XFAD male mice treated with TBZ were higher than in 5XFAD male mice treated with water. These results could indicate that this drug, rather than decreasing the levels of Aβ, increased the levels of Aβ in the male brain. Moreover, TBZ is shown to have inverse effects in male and female mice, perturbing in the correct direction the female mice. This is an indication that TBZ could cause Aβ accumulation for this specific dose/administration plan in male mice. These results are also in agreement with the in vitro inhibition assay of BACE1, in which we can observe an increase in enzyme when using TBZ, leading to Aβ accumulation. However, further investigation with more samples and additional concentrations would be necessary before excluding TBZ from a potential AD treatment.

The trend showing that male 5XFAD treated with water exhibited lower levels of Aβ compared to female 5XFAD treated with water is supported by a study in 2013 [25], which has reported that female 5XFAD mice had higher levels of Aβ than male mice, and this trend related to sex became more apparent as the mice aged. Moreover, Oblak and colleagues [40] detected 11% of the total variance in female and male 5XFAD mice by principal component analysis of transcriptomics data, results that suggest the presence of sex-biased molecular changes in animals. Lastly, a study in 2021 [41] showed sex differences in female and male 5XFAD mice, with females showing higher Aβ40 and 42 and developing pathology with early onset. These higher levels of Aβ females are probably explained by the presence of an estrogen response element in the Thy1 promoter, which drives the transgene expression in the specific model and hence causes increased Aβ [24,25,41].

As supported both from our initial computational work [13] and from the experimental work carried out here, Pik-90 shows very promising results. It is an experimental drug and hence has not been studied extensively; it requires further investigation in its role for AD. This output is supported both by the bioinformatics functional analysis carried out here, where a high number of shared pathways were detected with the AD-associated pathways from DisGeNET, as well as from the PPI network and the indirect route to BACE1.

## 5. Conclusions

Overall, our study concluded that out of the 13 chemical compounds tested, 2 drugs showed the most interesting effects: clomiphene citrate and Pik-90. Moreover, TBZ did not show any significant effect as a potential treatment for AD. However, further research is needed on these two drugs. In the future, we plan to extend this work through testing different concentrations of the drugs in assay experiments to detect the most effective concentration. Once the optimal concentration is selected, each drug could be tested in the appropriate AD cell model to confirm its effect. One such example is the use of iPSC derived cells in vitro, which fully recapitulate AD. Additionally, the results carried out in the mice are also preliminary, and treatment was carried out in a single concentration with the mice sample size being very small. Therefore, the experiment could be repeated using a larger sample and testing different concentrations of the compounds. However, through this experimental work and through the small-scale bioinformatics investigation that followed these results, we were able to provide some preliminary information regarding the function of clomiphene citrate and Pik-90 in vitro, and the function of TBZ in vivo, as well as to provide data showing the opposite effects of camptothecin and gatifloxacin in the assays. Finally, this study gave us the opportunity to validate some of our computational outputs from our previous computational work on network-based stage-specific drug repurposing in AD [13], giving feedback and strengthening our computational repurposing approaches.

## Figures and Tables

**Table 1:** The 13 drugs that were included in our study, the Braak stage they were detected in our previous work [13], and their current main indication.

Drug	AD Braak Stage	Main Indication
Tetrabenazine	V–VI	Huntington’s disease symptoms and other hyperkinetic movement disorders
Clomiphene citrate	I–II	Infertility
Camptothecin	I–II	Cancer treatment
Homoharringtonine	III–IV	Chronic myeloid leukaemia
Paroxetine	III–IV	Major depressive disorder, obsessive-compulsive disorder, social anxiety disorder, panic disorder, posttraumatic stress disorder, generalized anxiety disorder and premenstrual dysphoric disorder
	V–VI	
Gatifloxacin	III–IV	Bacterial infections
Iloperidone	V–VI	Schizophrenia
BI-2536	V–VI	Experimental drug
Piperidolate hydrochloride	III–IV	Convulsive pain in gastrointestinal diseases
	V–VI	
Emetine (hydrochloride hydrate)	I–II	Emesis induction in acute oral poisonings
	III–IV	
Pik-90	I–II	Experimental drug
Scoulerine	V–VI	Experimental drug
Ro-04-5595	III–IV	Experimental drug

**Table 2:** Methods for statistical analysis of experiments. (b&a; before and after), (a; after).

Section	Analysis	Categories	Statistical Methods
Effect of tested compounds on BACE1 enzyme activity	Comparison of the response of the top 13 drugs against IC and EC	Clomiphene Citrate TBZ Camptothecin Homoharringtonine Paroxetine Gatifloxacin Iloperidone BI-2536 Piperidolate HCl Pik-90 Scoulerine Ro-04-5595 IC EC	One-Way ANOVA with Bonferroni multiple comparison
	Comparison of AUC means overall inhibition of the clomiphene citrate and Pik-90 against IC and EC	Clomiphene Citrate TBZ IC EC	One-Way ANOVA with Bonferroni multiple comparison
Y-maze test before and after tetrabenazine treatment in 5XFAD mice	Comparison of the performance in Y-maze before and after treatment within the same group and across different groups	FAD H_2_O F (b&a) FAD TBZ F (b&a) FAD H_2_O M (b&a) FAD TBZ M (b&a) WT H_2_O F (b&a) WT TBZ F (b&a) WT H_2_O M (b&a) WT TBZ M (b&a)	Paired (between the same group) and non-paired *t*-test (between different groups)
ELISA test of Aβ peptide in 5XFAD mice	Comparison of Aβ levels in 5XFAD and WT mice, both treated with TBZ and water	FAD H_2_O F (a) FAD TBZ F (a) FAD H_2_O M (a) FAD TBZ M (a) WT H_2_O F (a) WT TBZ F (a) WT H_2_O M (a) WT TBZ M (a)	Unpaired two samples Wilcoxon test

## Data Availability

Not applicable.

## Supplementary Materials

The following supporting information can be downloaded at: https://www.mdpi.com/article/10.3390/life13051095/s1, Table S1: Commonalities of the Biological Processes of targets of interest.
